# Regulation of CYP27B1 mRNA Expression in Primary Human Osteoblasts

**DOI:** 10.1007/s00223-016-0131-9

**Published:** 2016-03-25

**Authors:** K. van der Meijden, H. W. van Essen, F. W. Bloemers, E. A. J. M. Schulten, P. Lips, N. Bravenboer

**Affiliations:** Department of Internal Medicine/Endocrinology, VU University Medical Center, Research Institute MOVE, Amsterdam, The Netherlands; Department of Clinical Chemistry, VU University Medical Center, Research Institute MOVE, PO Box 7057, 1007 MB Amsterdam, The Netherlands; Department of Trauma Surgery, VU University Medical Center, Amsterdam, The Netherlands; Department of Oral and Maxillofacial Surgery/Oral Pathology, VU University Medical Center, Academic Centre for Dentistry Amsterdam (ACTA), Amsterdam, The Netherlands

**Keywords:** CYP27B1, Primary human osteoblasts, Calcium, Parathyroid hormone, Fibroblast growth factor 23, Matrix extracellular phosphoglycoprotein

## Abstract

The enzyme 1α-hydroxylase (gene CYP27B1) catalyzes the synthesis of 1,25(OH)_2_D in both renal and bone cells. While renal 1α-hydroxylase is tightly regulated by hormones and 1,25(OH)_2_D itself, the regulation of 1α-hydroxylase in bone cells is poorly understood. The aim of this study was to investigate in a primary human osteoblast culture whether parathyroid hormone (PTH), fibroblast growth factor 23 (FGF23), calcitonin, calcium, phosphate, or MEPE affect mRNA levels of CYP27B1. Our results show that primary human osteoblasts in the presence of high calcium concentrations increase their CYP27B1 mRNA levels by 1.3-fold. CYP27B1 mRNA levels were not affected by PTH1-34, rhFGF23, calcitonin, phosphate, and rhMEPE. Our results suggest that the regulation of bone 1α-hydroxylase is different from renal 1α-hydroxylase. High calcium concentrations in bone may result in an increased local synthesis of 1,25(OH)_2_D leading to an enhanced matrix mineralization. In this way, the local synthesis of 1,25(OH)_2_D may contribute to the stimulatory effect of calcium on matrix mineralization.

**Electronic supplementary material:**

The online version of this article (doi:10.1007/s00223-016-0131-9) contains supplementary material, which is available to authorized users.

## Introduction

The calciotropic hormone 1,25-dihydroxyvitamin D (1,25(OH)_2_D) is synthesized by the mitochondrial enzyme 25-hydroxyvitamin D-1α-hydroxylase (1α-hydroxylase) encoded by the gene CYP27B1 [33]. The enzyme 1α-hydroxylase is predominantly expressed in the proximal tubular cells of the kidney which is the major source of circulating 1,25(OH)_2_D [11]. Extra-renal sites of 1α-hydroxylase expression such as bone cells are responsible for the local synthesis of 1,25(OH)_2_D [2]. Both renal and bone cells express identical 1α-hydroxylase proteins, however, the regulation of 1α-hydroxylase at these sites is different [3, 4, 26]. While renal 1α-hydroxylase is tightly regulated by hormones and 1,25(OH)_2_D itself [61], the regulation of 1α-hydroxylase in bone cells is poorly understood.

Renal 1α-hydroxylase expression and activity are strictly regulated by the hormones parathyroid hormone (PTH) and fibroblast growth factor 23 (FGF23) [61]. PTH is secreted by the parathyroid glands in response to low serum calcium concentrations and induces renal CYP27B1 expression [19]. FGF23 is secreted by osteocytes and osteoblasts in response to phosphate loading or high serum 1,25(OH)_2_D concentrations [41]. FGF23 mainly acts on the kidney by inhibiting renal tubular phosphate reabsorption, but it also suppresses renal CYP27B1 expression [41, 47]. Direct effects of calcium on CYP27B1 expression have also been reported [9, 19]. A low calcium concentration increases CYP27B1 expression and a high calcium concentration reduces CYP27B1 expression in transformed human proximal tubule cells and primary mouse kidney cells [9, 19] Whether phosphate directly regulates CYP27B1 expression in renal cells remains controversial. In primary mouse kidney cells, low phosphate conditions increase the activity of 1α-hydroxylase [14, 19], but direct effects of phosphate have not been confirmed by other investigators [24, 25, 45]. Another hormone involved in the regulation of 1α-hydroxylase may be calcitonin, which is synthesized by C-cells of the thyroid gland [43]. Although in humans the role of calcitonin in the regulation of 1α-hydroxylase has been questioned [27], several animal studies show stimulatory effects of calcitonin on renal CYP27B1 expression [28, 31, 43, 52].

Previously, we revealed that in primary human osteoblasts 1,25(OH)_2_D did not affect CYP27B1 expression [58], suggesting that bone 1α-hydroxylase is not regulated as renal 1α-hydroxylase. However, whether the other renal regulators can affect bone 1α-hydroxylase has not been fully elucidated. PTH exerts its effects through binding to the PTH receptor 1 (PTH1R) which is present in osteoblasts [53], but effects of PTH on CYP27B1 expression seem to be controversial. In the human osteoblast cell line SV-HFO and rat osteosarcoma cell line ROS 17/2.8, PTH does not regulate expression and activity of CYP27B1 [55, 59]. However, in primary cells including human bone cells and mesenchymal stem cells a stimulatory effect of PTH has been shown on 1α-hydroxylase expression and activity [21, 53]. FGF23 can inhibit extra-renal expression of CYP27B1 as shown in monocyte cultures [7], but whether FGF23 modulates CYP27B1 expression in bone cells is unknown. Due to undetectable Klotho mRNA levels in bone cells, FGF23 can bind fibroblast growth factor receptor (FGFR) only with low affinity [56]. Nevertheless, there is evidence that supra-physiological concentrations of FGF23 are able to affect bone cells [63]. Calcium does not appear to modulate 1α-hydroxylase expression and activity in SV-HFO osteoblasts [59]. Phosphate increases CYP27B1 mRNA expression in the mouse IDG-SW3 cell line [29]. Whether calcium or phosphate affects CYP27B1 expression in primary human bone cells is unknown. Regarding calcitonin, primary human osteoblasts express the calcitonin receptor (CTR) [62], but effects of calcitonin on the expression of CYP27B1 in bone cells have not yet been investigated.

Due to the local function of 1,25(OH)_2_D synthesis in bone, for instance stimulation of osteoblast differentiation and mineralization in an autocrine or paracrine way [5, 58, 59], we hypothesized that the enzyme 1α-hydroxylase is regulated at a local level. A factor that may be involved in this regulation of local 1,25(OH)_2_D concentrations in bone is matrix extracellular phosphoglycoprotein (MEPE). MEPE is a member of the Small Integrin Binding Ligand N-linked Glycoprotein (SIBLING) family and is predominantly expressed in osteocytes and osteoblasts [44, 46]. Animal studies show that MEPE inhibits renal phosphate reabsorption and reduces intestinal phosphate absorption [15, 39]. Similar to FGF23, MEPE also inhibits bone mineralization [23, 63]. In ex vivo cultures of MEPE knockout mouse osteoblasts, an increased amount of mineralized nodules was observed compared to wild-type osteoblast cultures [23]. Bone mineralization is also modulated by locally synthesized 1,25(OH)_2_D as shown in vitro [5, 59]. Synthesis of 1,25(OH)_2_D from 25(OH)D by osteoblasts in culture leads to an increased matrix mineralization [5, 59]. Because 1,25(OH)_2_D and MEPE are both involved in bone mineralization [5, 23, 59], MEPE may be able to regulate CYP27B1 expression in bone. Due to the stimulatory effect of 1,25(OH)_2_D on matrix mineralization [5, 59], we hypothesized that MEPE acts as an inhibitor of bone CYP27B1.

The aim of the present study was to investigate in a primary human osteoblast culture whether PTH, FGF23, calcitonin, calcium, phosphate, or MEPE affect mRNA levels of CYP27B1. Since the function of extra-renal synthesis of 1,25(OH)_2_D is different from that of the renal synthesis of 1,25(OH)_2_D [5, 59], we hypothesized that the renal regulators do not affect CYP27B1 mRNA levels in primary human osteoblasts. Regarding MEPE, we hypothesized that it reduces mRNA levels of CYP27B1. Because the impact of locally synthesized 1,25(OH)_2_D on bone cells not only depends on the concentration, but also on expression levels of the vitamin D receptor (VDR), we determined VDR mRNA levels in primary human osteoblasts as well.

## Materials and Methods

### Primary Human Osteoblast Culture

Primary human osteoblasts were isolated from redundant trabecular bone fragments obtained from healthy donors undergoing pre-implant bony reconstruction of the mandible or maxilla with autologous bone from the anterior iliac crest. Trabecular bone fragments were also obtained from femoral heads from patients who underwent orthopedic surgery for fractures of the femoral neck. The donor group consisted of 10 males and 7 females with a mean age of 56.2 ± 4.6 years. The protocol was approved by the Medical Ethical Review Board of the VU University Medical Center, Amsterdam, The Netherlands, and all donors gave their written informed consent.

A modification of the methods of Beresford et al. and Marie et al. [8, 37] was used to obtain a primary human osteoblast culture, as described previously [58]. Shortly, the trabecular bone fragments were minced into small pieces and washed extensively with phosphate-buffered saline (PBS). The bone pieces were treated with 2 mg/ml collagenase type II (300 U/mg; Worthington Biochemical Corporation, Lakewood, NJ, USA) for 2 h in a shaking waterbath at 37 °C. The pieces were placed in culture flasks with Dulbecco’s Modified Eagle Medium: Nutrient Mixture F-12 (DMEM/F12; Gibco, Life technologies, Grand Island, NY, USA) supplemented with 10 % Fetal Clone I (HyClone; Thermo Fisher Scientific, Rockford, IL, USA), 100 U/ml penicillin and 100 μg/ml streptomycin (Gibco; Life technologies), 1.25 μg/ml fungizone (Gibco; Life technologies) and incubated at 37 °C in a humidified air with 5 % CO_2_. Medium was changed twice a week until cells reached confluence.

### Primary Human Osteoblast Treatments

Primary human osteoblasts of the first or second passage were seeded into a 12-wells plate at a cell density of 40.000 cells/well. Cells were allowed to attach to the well for 24 h. Subsequently, primary human osteoblasts were incubated in medium supplemented with human PTH fragment 1-34 (PTH1-34; Sigma-Aldrich, St. Louis, MO, USA), recombinant human FGF23 protein (rhFGF23; R&D Systems, Minneapolis, MN, USA), human calcitonin (Sigma-Aldrich), calcium chloride (CaCl_2_; Sigma-Aldrich), sodium dihydrogen phosphate (NaH_2_PO_4_; Merck, Darmstadt, Germany), or recombinant human MEPE (rhMEPE; R&D systems). Cells were treated with four different concentrations ranging from physiological to supra-physiological of PTH (50–50,000 pg/ml), FGF23 (25–25,000 pg/ml), and calcitonin (2–2000 pg/ml). Three different concentrations of rhMEPE (0.05-5 μg/ml; physiological concentrations of MEPE: 0.02–1.3 μg/ml) were used, as published previously [32]. Supplementation of four different concentrations of calcium (0.5–3.0 mmol/l) was performed in DMEM without calcium (Gibco; Life technologies). Supplementation of four different concentrations of phosphate (0.5–3.0 mmol/l) was performed in DMEM without phosphate (Gibco; Life technologies). Incubation of primary human osteoblasts in PTH1-34, rhFGF23, calcitonin, or rhMEPE was performed in DMEM/F12 containing 1.1 mmol/l calcium and 1.0 mmol/l phosphate. All experiments were performed in medium with 5 % Fetal Clone I. After 24 h of incubation, cells were lysed for total RNA isolation as described below.

### RNA Isolation and RT-qPCR

Total RNA isolation of primary human osteoblasts was performed using the RNeasy Mini Kit (Qiagen, Hilden, Germany) according to the manufacturer’s protocol. For removing residual DNA amounts, an additional on-column DNAse treatment was accomplished during the RNA isolation procedure. Total RNA concentrations were measured by the Nanodrop spectrophotometer (Nanodrop Technologies, Wilmington, DE, USA).

RNA was reverse transcribed from 100 ng total RNA in a 20-μl reaction mixture containing 5 mmol/l MgCl_2_ (Eurogentec, Maastricht, The Netherlands), 1x RT buffer (Promega, Madison, WI, USA), 1 mmol/l dATP, 1 mmol/l dCTP, 1 mmol/l dGTP, 1 mmol/l dTTP (Roche Diagnostics, Mannheim, Germany), 1 mmol/l betaïne, 10 ng/ul random primer, 0.4 U/μl RNAsin (Promega), and 5 U/μl M-MLV RT-enzyme (Promega), as described previously [58]. The PCR reaction of total 25 μl contained 3 μl cDNA, 300 nmol/l reverse and forward primer, and SYBR Green Supermix (Bio-Rad Laboratories Inc., Veenendaal, The Netherlands). The following primer sets were used: CYP27B1 forward: 5′-TGGCCCAGATCCTAACACATTT-3′, reverse: 5′-GTCCGGGTCTTGGGTCTAACT-3′; VDR forward: 5′-GGACGCCCACCATAAGACCTA-3′, reverse: 5′-CTCCCTCCACCATCATTCACA-3′; osterix forward: 5′-TACCCCATCTCCCTTGACTG-3′, reverse: 5′-TCTCCATAACCATGGCAACA-3′; runt-related transcription factor 2 (RUNX2) forward: 5′-CGCATTCCTCATCCCAGTAT-3′, reverse: 5′-GCC-TGG-GGT-CTG-TAA-TCT-GA-3′; collagen type 1α1 (COL1α1) forward: 5′-GTGCTAAAGGTGCCAATGGT-3′, reverse: 5′-ACCAGGTTCACCGCTGTTAC-3′, alkaline phosphatase (ALP) forward: 5′-CCACGTCTTCACATTTGGTG-3′, reverse: 5′-GCAGTGAAGGGCTTCTTGTC-3′; osteopontin forward: 5′-TTCCAAGTAAGTCCAACGAAAG-3′, reverse: 5′- GTGACCAGTTCATCAGATTCAT-3′; osteocalcin forward: 5′-GCGCTACCTGTATCAATGGTATA-3′, reverse: 5′-TCAGCCAACTCGTCACAGTC-3′; fibroblast growth factor 23 (FGF23) forward: 5′-TGAGCGTCCTCAGAGCCTAT-3′, reverse: 5′-TTGTGGATCTGCAGGTGGTA-3′; dentin matrix protein 1 (DMP1) forward: 5′-GATCAGCATCCTGCTCATGTT-3′, reverse: 5′-AGCCAAATGACCCTTCCATTC-3′ [6]; SOST forward: 5′- ACCACCCCTTTGAGACCAAAG-3′, reverse: 5′-GGTCACGTAGCGGGTGAAGT-3′ [6]; calcium-sensing receptor (CaSR) forward: 5′-TCAACCTGCAGTTCCTGCTGG-3′, reverse: 5′-TGGCATAGGCTGGAATGAAGG-3′ [30]; TATA-binding protein (TBP) forward: 5′-GGTCTGGGAAAATGGTGTGC-3′, reverse: 5′-GCTGGAAAACCCAACTTCTG-3′. The PCR was performed on an iCycler iQ™ Real-Time PCR Detection System (Bio-Rad): 3 min at 95 °C, 40 cycles consisting of 15 s at 95 °C and 1 min at 60 °C. The relative gene expression was calculated by the 2^−ΔCt^ method and TBP as well as succinate dehydrogenase subunit A (SDHA; Primerdesign, Rownhams, Southampton, United Kingdom) were used as reference genes.

### Statistical Analysis

Data are presented as mean ± standard error of the mean (SEM). Of each factor a dose–response was tested using Friedman test followed by Dunn’s post hoc test. A *p* value <0.05 was considered to be statistically significant. Data were analyzed using GraphPad Prism 4 (Graphpad Software, San Diego, CA, USA).

## Results

### Effects of PTH1-34, rhFGF23, or Calcitonin on mRNA Levels of CYP27B1 and VDR in Primary Human Osteoblasts

**Fig. 1:**
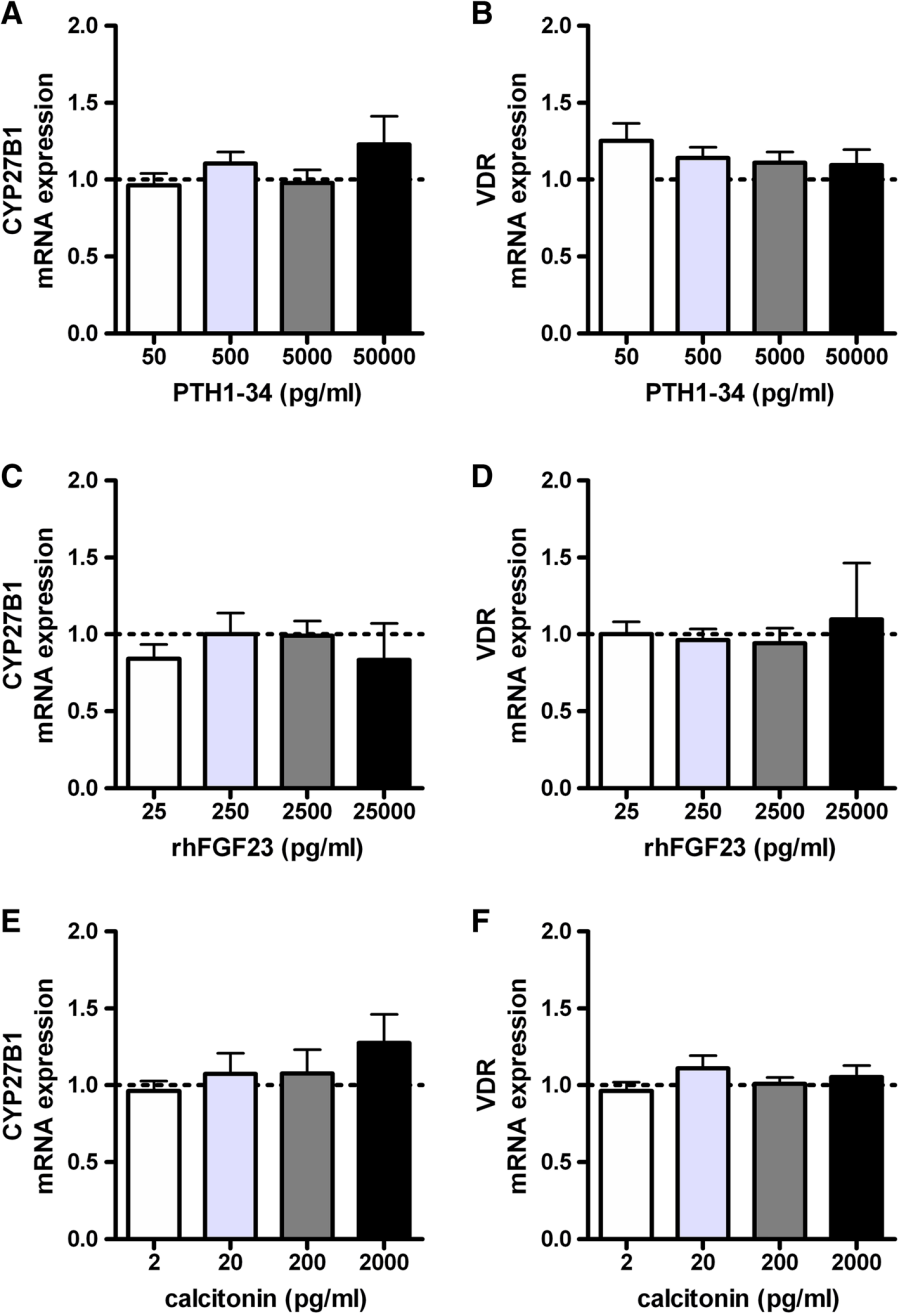
Effects of PTH1-34, rhFGF23, and calcitonin on mRNA levels of CYP27B1 and VDR in primary human osteoblasts. CYP27B1 and VDR mRNA levels were determined after 24 h incubation of primary human osteoblasts in medium supplemented with increasing concentrations of PTH1-34 (**a** and **b**, respectively), rhFGF23 (**c** and **d**, respectively), or calcitonin (**e** and **f**, respectively). Results (mean ± SEM) are expressed as treatment versus control ratios (control was set at 1.0; *dashed line*) using cells from 4 to 6 different donors

Increasing concentrations of PTH1-34 (50–50,000 pg/ml) did not affect mRNA levels of CYP27B1 or VDR (Fig. 1a, b, respectively). Increasing concentrations of rhFGF23 (25-25,000 pg/ml) did also not affect mRNA levels of CYP27B1 and VDR (Fig. 1c, d, respectively), nor did calcitonin (2–2000 pg/ml; Fig. 1e, f, respectively).

### Effects of Calcium or Phosphate on mRNA Levels of CYP27B1 and VDR in Primary Human Osteoblasts

**Fig. 2:**
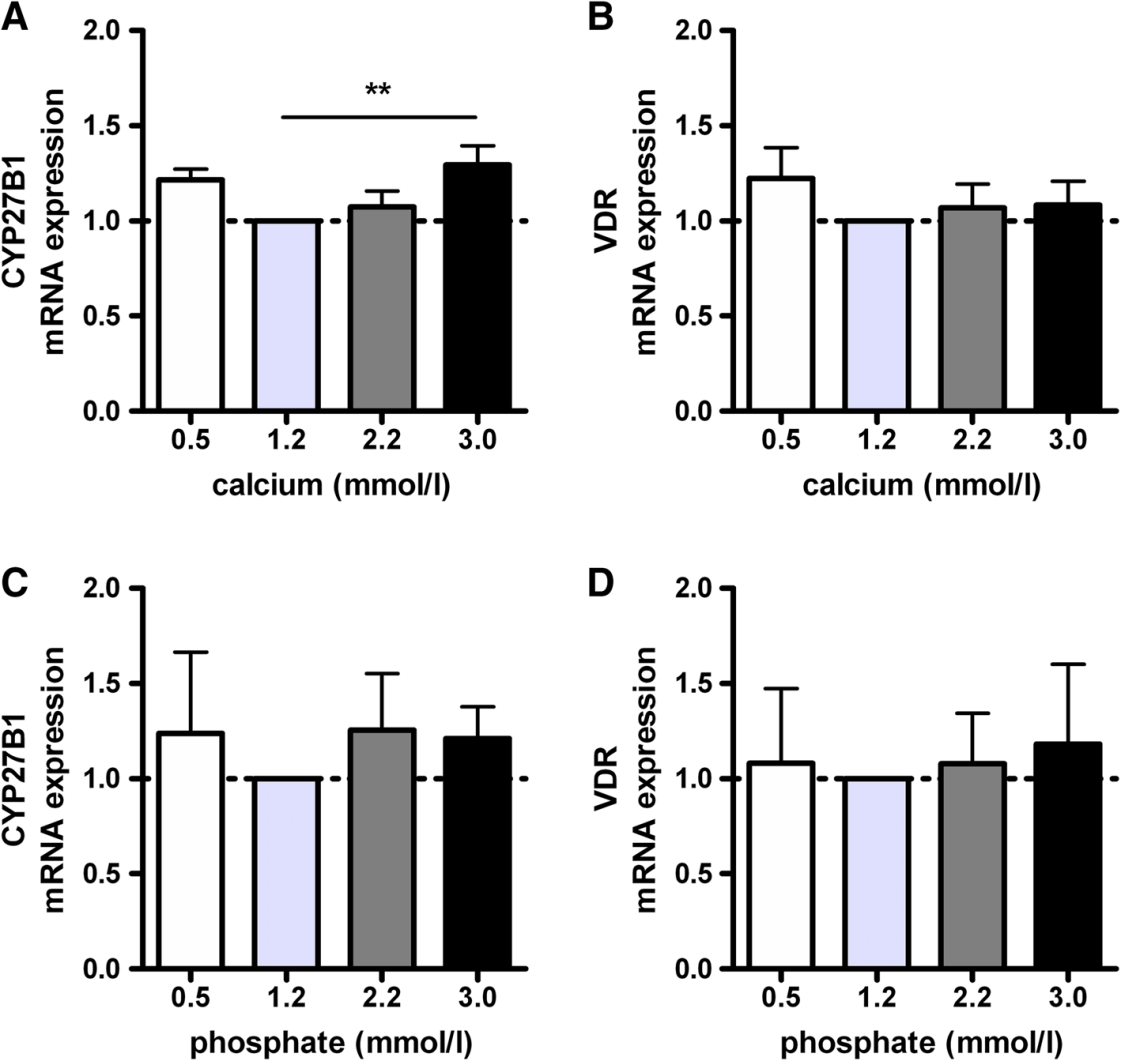
Effects of calcium and phosphate on mRNA levels of CYP27B1 and VDR in primary human osteoblasts. CYP27B1 and VDR mRNA levels were determined after 24 h incubation of primary human osteoblasts in medium supplemented with increasing concentrations of calcium (**a** and **b**, respectively) or phosphate (**c** and **d**, respectively). Results (mean ± SEM) are expressed as treatment versus control ratios (control was set at 1.0; *dashed line*) using cells from 5 different donors. (**p < 0.01)

Calcium and phosphate concentrations of 1.2 mmol/l were used as control because these concentrations closely resemble the concentrations of calcium and phosphate in DMEM/F12 supplemented with fetal bovine serum. High calcium concentrations increased CYP27B1 mRNA levels by 1.3-fold (*p* < 0.01; Fig. 2a), but an effect of low or high calcium concentrations on VDR mRNA levels was not observed (Fig. 2b). CYP27B1 and VDR mRNA levels were not affected by different concentrations of phosphate in medium (Fig. 2c, d, respectively).

### Effects of Calcium on mRNA Levels of Differentiation Markers in Primary Human Osteoblasts

**Fig. 3:**
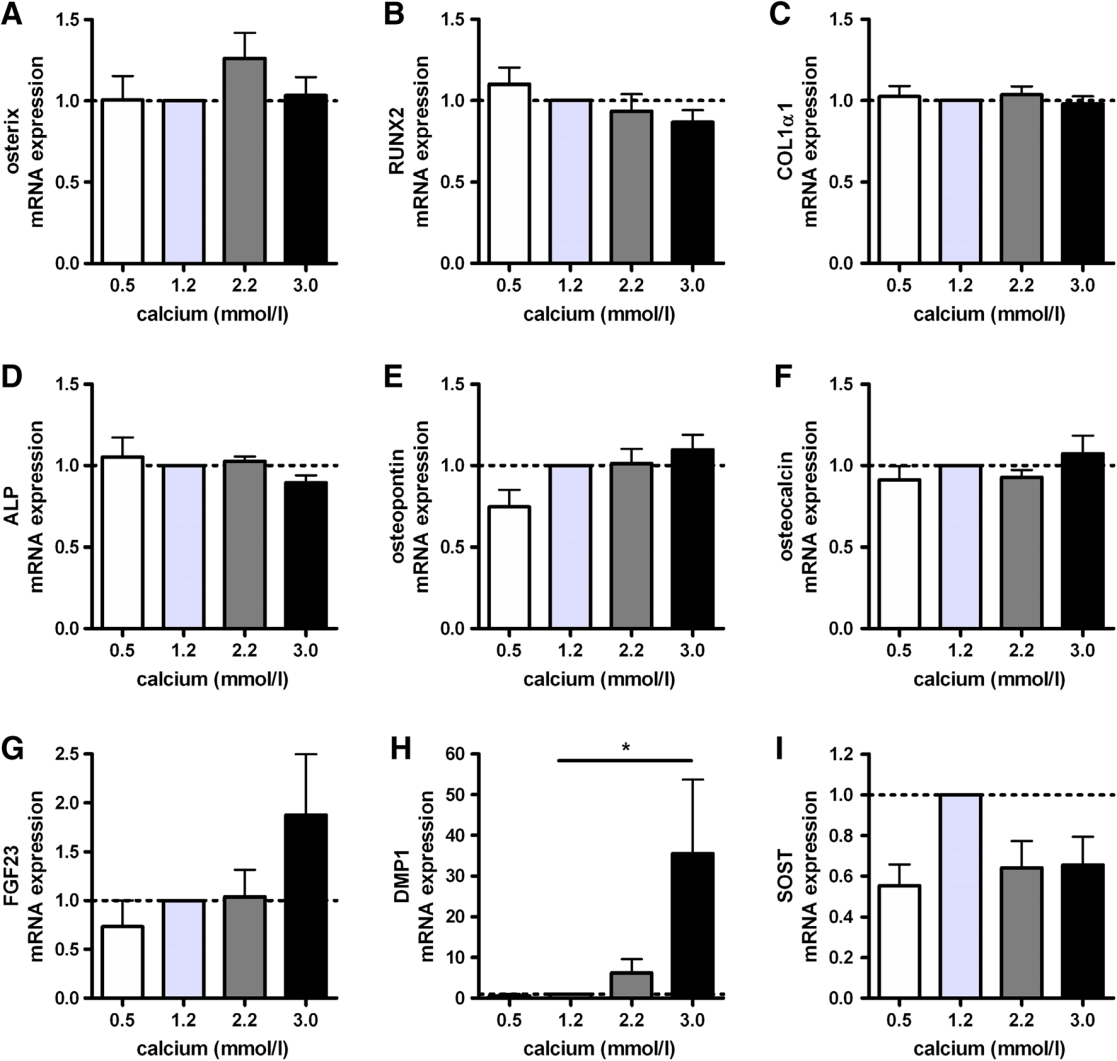
Effects of calcium on differentiation markers in primary human osteoblasts. Osterix (**a**), RUNX2 (**b**), COL1α1 (**c**), ALP (**d**), osteopontin (**e**), osteocalcin (**f**), FGF23 (**g**), DMP1 (**h**), and SOST (**i**) mRNA levels were determined after 24 h incubation of primary human osteoblasts in medium supplemented with increasing concentrations of calcium. Results (mean ± SEM) are expressed as treatment versus control ratios (control was set at 1.0; *dashed line*) using cells from 5 different donors. (*p < 0.05)

Because it has been shown that high calcium concentrations stimulate osteoblast differentiation and that more differentiated osteoblasts have a higher CYP27B1 activity [3, 35], we analyzed mRNA levels of several differentiation markers after incubation of osteoblasts in medium supplemented with increasing concentrations of calcium (Figs. 3a–i). High calcium concentrations increased mRNA levels of dentin matrix protein 1 (DMP1) by 35.5-fold (*p* < 0.05). Osterix, runt-related transcription factor 2 (RUNX2), collagen type 1α1 (COL1α1), alkaline phosphatase (ALP), osteopontin, osteocalcin, FGF23, and SOST mRNA levels were not affected by increasing concentrations of calcium.

### Effects of Calcium on mRNA Levels of the Calcium Sensing Receptor (CaSR) in Primary Human Osteoblasts

**Fig. 4:**
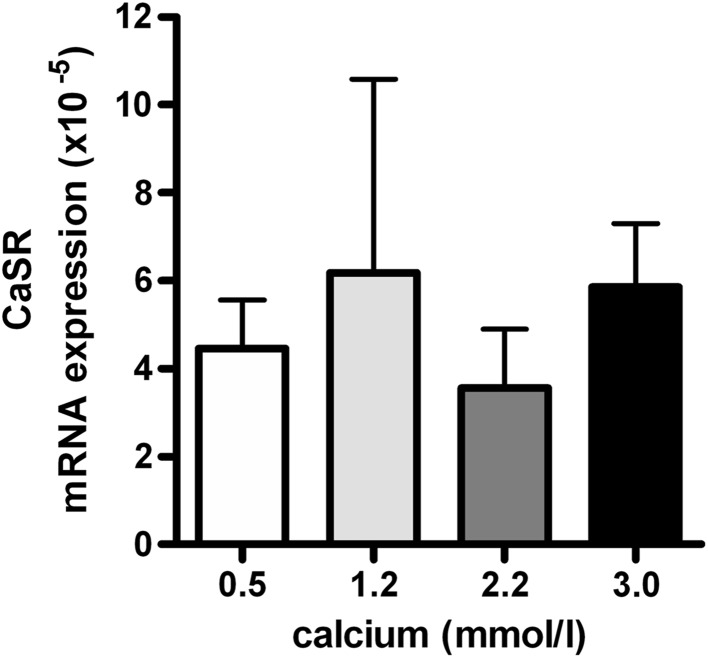
Effects of calcium on CaSR mRNA levels in primary human osteoblasts. CaSR mRNA levels were determined after 24 h incubation of primary human osteoblasts in medium supplemented with increasing concentrations of calcium. Results are expressed as mean ± SEM using cells from 5 different donors

Primary human osteoblasts expressed extremely low CaSR mRNA levels (Fig. 4). Increasing concentrations of calcium did also not stimulate mRNA levels of CaSR.

### Effects of MEPE on mRNA Levels of CYP27B1 and VDR in Primary Human Osteoblasts

**Fig. 5:**
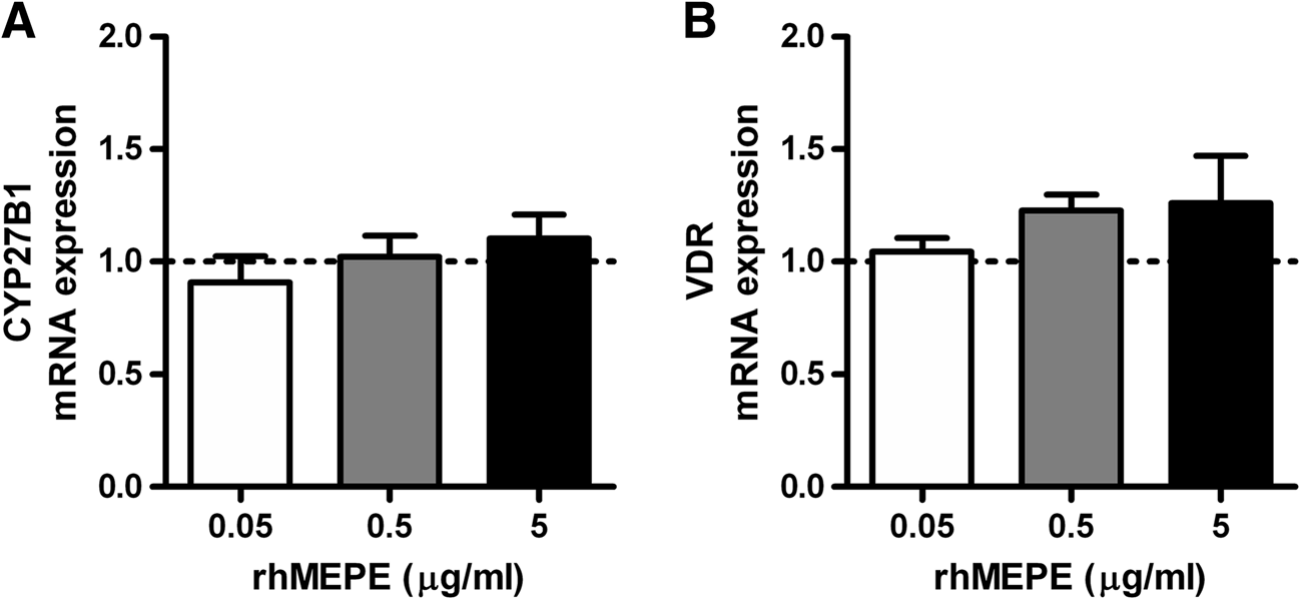
Effects of rhMEPE on mRNA levels of CYP27B1 and VDR in primary human osteoblasts. CYP27B1 and VDR mRNA levels were determined after 24 h incubation of primary human osteoblasts in medium supplemented with increasing concentrations of rhMEPE (**a** and **b**, respectively). Results (mean ± SEM) are expressed as treatment versus control ratios (control was set at 1.0; *dashed line*) using cells from 5 different donors

Increasing concentrations of rhMEPE (0.05-5 ug/ml) did not affect mRNA levels of CYP27B1 and VDR (Fig. 5a, b, respectively).

## Discussion

The enzyme 1α-hydroxylase catalyzes the synthesis of 1,25(OH)_2_D in both renal and bone cells [2, 11]. While renal 1α-hydroxylase is tightly regulated by hormones and 1,25(OH)_2_D itself [61], the regulation of 1α-hydroxylase in bone cells is poorly understood. We hypothesized that all renal regulators did not affect CYP27B1 mRNA levels. In contrast to our hypothesis, we observed that primary human osteoblasts in the presence of high calcium concentrations increase their CYP27B1 mRNA levels. Thus, calcium appears to play a role in the regulation of 1α-hydroxylase in both kidney and bone tissue. However, high serum calcium concentrations reduce CYP27B1 expression levels in the kidney [9], while we observed the opposite effect in bone cells.

Changes in extracellular calcium concentrations can occur during bone remodeling [20]. In vitro studies show that human osteoblasts respond to high calcium concentrations by an increased chemotaxis and proliferation [12, 22, 36, 48]. High extracellular calcium concentrations also lead to stimulation of osteoblast differentiation markers as shown in fetal rat calvarial cells [16], and an enhanced formation of mineralized nodules [16, 64]. In late mature and mineralizing cultures of primary mouse osteoblasts, the CYP27B1 activity is higher compared to less differentiated osteoblasts [3]. Therefore, we hypothesized that primary human osteoblasts in our study were stimulated to differentiate in the presence of high calcium concentrations resulting in higher CYP27B1 expression. Indeed, high calcium concentrations increased mRNA levels of DMP1 which is a marker of the osteocyte [10]. As the cells used in our study were mature osteoblasts (Supplementary Fig.), the high calcium concentrations may have stimulated the osteoblasts in culture to differentiate. Thus, the increased expression of CYP27B1 in the presence of high calcium is most likely a result of an increased maturation state.

The mechanism by which bone cells sense changes in extracellular calcium has been reported to occur through the CaSR, which is a member of the guanine nucleotide-binding protein (G-protein)-coupled receptor (GPCR) family [13]. Studies using fetal rat calvarial cells and clonal murine osteoblast cells suggest that the CaSR is involved in the stimulatory effects of calcium on osteoblast differentiation since these effects are mimicked by CaSR agonists [16]. Moreover, abolition of the CaSR reduces osteoblast differentiation and mineralization in mouse MC3T3-E1 cells [64]. This suggests that the increase of DMP1 mRNA levels under high calcium conditions may have occurred at least in part through activation of the CaSR. In our study, however, primary human osteoblasts expressed extremely low CaSR mRNA levels, even in the presence of high calcium concentrations. This finding raises the question whether other calcium sensing mechanisms may be involved [38, 49].

Increased CYP27B1 mRNA levels in osteoblasts under high calcium conditions may have positive effects on bone, because increased CYP27B1 expression levels may lead to higher 1,25(OH)_2_D concentrations locally. High 1,25(OH)_2_D concentrations stimulate not only osteoblast differentiation, but also mineralization [5, 59] which is possible in the presence of high calcium. High calcium intake by rats also leads to increased mRNA levels of CYP27B1 in bone compared to rats with a low calcium intake [3]. CYP24 mRNA levels were also higher in bones from rats with a high calcium intake compared to rats fed a low calcium diet [3], suggesting that 1,25(OH)_2_D concentrations in bone tissue were higher in rats fed a high calcium diet [3, 42]. Thus, the increased synthesis of 1,25(OH)_2_D by bone cells under high calcium conditions may contribute at least partially to the stimulatory effect of calcium on matrix mineralization, as proposed previously [42].

The availability and the impact of locally synthesized 1,25(OH)_2_D not only depend on the activity of 1α-hydroxylase, but also on the expression of VDR and the activity of 24-hydroxylase. In our study, CYP24 mRNA levels were extremely low or even undetectable in some donors (data not shown). VDR mRNA levels were not affected by increased concentrations of calcium, suggesting that the response of osteoblasts to 1,25(OH)_2_D is unchanged under high calcium conditions.

PTH exerts its effects through binding to the PTH receptor 1 (PTH1R) leading to actions to regulate bone remodeling [53]. In our study, PTH1-34 did not stimulate the expression of CYP27B1 which is in line with other in vitro studies using ROS and SV-HFO osteoblasts [55, 59]. In addition to in vitro studies, PTH does also not stimulate CYP27B1 mRNA levels in vivo [3]. Our results are in contrast to a study performed in human mesenchymal stem cells in which PTH1-34 increased CYP27B1 expression and 1,25(OH)_2_D synthesis [21]. This suggests a different regulation of CYP27B1 in osteoblasts compared to mesenchymal stem cells which seems to depend on the maturation state [60]. Another study in primary human bone cells showed that PTH1-84 increased CYP27B1 mRNA expression and 1,25(OH)_2_D synthesis, but concentrations of PTH1-84 (66 nmol/l) in that study were much higher compared to our study [54].

The primary role of calcitonin is to inhibit osteoclast activity leading to reduced bone resorption [27], but several studies have shown that calcitonin also affects osteoblast function [17, 18]. Osteoblast proliferation and alkaline phosphatase activity increase in the presence of calcitonin [17, 18]. Thus calcitonin is able to affect osteoblast proliferation and differentiation, but our results suggests that the local synthesis of 1,25(OH)_2_D is not involved in the actions of calcitonin.

Changes in phosphate concentrations did not appear to affect CYP27B1 mRNA levels in primary human osteoblasts. This is in contrast to a study in mouse osteocytes in which high phosphate concentrations (4 and 10 mmol/l) stimulate CYP27B1 mRNA levels dose-dependently [29], but those phosphate concentrations were much higher than we used in our study. Regarding FGF23, effects of high concentrations have been shown on bone cells in vitro despite undetectable Klotho mRNA levels [63]. In fetal rat calvarial cell cultures, overexpression of FGF23 leads to the suppression of osteoblast differentiation and matrix mineralization [63]. In our study, incubation of human osteoblasts in the presence of both physiological and supra-physiological concentrations of FGF23 did not result in altered CYP27B1 mRNA levels. This suggests that FGF23 does not regulate CYP27B1 expression in bone cells.

Consistent with previous animal and in vitro studies [3, 55, 59], our study suggests that CYP27B1 is regulated differently in bone compared with the kidney. Differences in regulation may be explained by differences in the contribution of repressor and enhancer elements in the 5′-flanking region of the CYP27B1 gene [55]. The difference in regulation of 1α-hydroxylase in bone tissue compared to renal 1α-hydroxylase possibly exists due to the differences in function. Locally synthesized 1,25(OH)_2_D does not function through endocrine pathways, but acts via autocrine and paracrine mechanisms to stimulate osteoblast differentiation and matrix mineralization [5, 58, 59]. To answer local demands, 1α-hydroxylase should be regulated at a local level. Local factors such as interleukin-1β and TGF-β, respectively, increase and decrease CYP27B1 expression levels in bone cells [55, 59]. Recently, it has been shown that mechanical loading also stimulates CYP27B1 mRNA levels in primary human osteoblasts [57]. Thus, the regulation of 1α-hydroxylase in bone appears to be tissue-specific.

We hypothesized that MEPE reduces CYP27B1 mRNA in human osteoblasts due to the inhibitory role of MEPE in bone mineralization. The inhibition of bone mineralization by MEPE has been related to the acidic serine- and aspartate-rich motif (ASARM) [34, 40, 50]. This small peptide is released after cleavage by cathepsin B and can bind to the hydroxyapatite crystal and, in turn, inhibit mineralization [1, 40]. The cleavage by cathepsin B can be prevented by an interaction of MEPE with PHEX [1]. In culture, ASARM peptides may be released after cleavage by cathepsin B which is expressed by osteoblasts [51]. Due to the overload of MEPE in culture, the capacity of expressed PHEX is most likely too low to prevent MEPE from cathepsin B cleavage, as suggested previously [51]. In our culture ASARM peptides could be present, but effects on CYP27B1 mRNA levels were not observed.

A limitation of this study is that only one time-point (24 h) was tested, while measurements of CYP27B1 mRNA levels on earlier or later time-points may give different results. The 24-h time-point was chosen based on other studies in which effects of factors such as interleukin-1β and TGF-β on CYP27B1 mRNA levels were also demonstrated after 24 h incubation [55, 59]. Another point that can be made is that the response of osteoblasts to the applied treatments may depend on the differentiation state. We used mature osteoblasts as determined by the measurement of mRNA levels of several differentiation markers (Supplementary Fig.), but it is possible that immature osteoblasts respond differently to treatments such as calcium and phosphate. Note, however, that increased CYP27B1 mRNA levels under high calcium conditions do not necessarily result in increased enzyme activity.

In conclusion, this in vitro study shows that MEPE as well as the renal regulators PTH, FGF23, phosphate, and calcitonin do not affect CYP27B1 mRNA levels in human bone cells. On the contrary, calcium positively affects CYP27B1 mRNA which suggests that the local synthesis of 1,25(OH)_2_D contributes to the stimulatory effect of calcium on matrix mineralization.

## Electronic supplementary material

Below is the link to the electronic supplementary material.

Supplementary material 1 (DOCX 245 kb)
