# Supplementary material for: Regulation of CYP27B1 mRNA Expression in Primary Human Osteoblasts
Source: Calcif Tissue Int. 2016 Mar 25;99:164–73. doi: 10.1007/s00223-016-0131-9 (PMC4932130; doi:10.1007/s00223-016-0131-9)
Supplement: Supplementary file 1 — Supplementary material 1 (DOCX 245 kb) [file 223_2016_131_MOESM1_ESM.docx]

**Supplementary figure. Differentiation state of primary human osteoblasts in culture.**

Osterix, RUNX2, COL1α1, ALP, osteopontin, osteocalcin, FGF23, DMP1 and SOST mRNA levels were determined in primary human osteoblasts cultured in medium without any treatments. Results are expressed as mean ± SEM using cells from 5 different donors.

**Supplementary figure.**

**
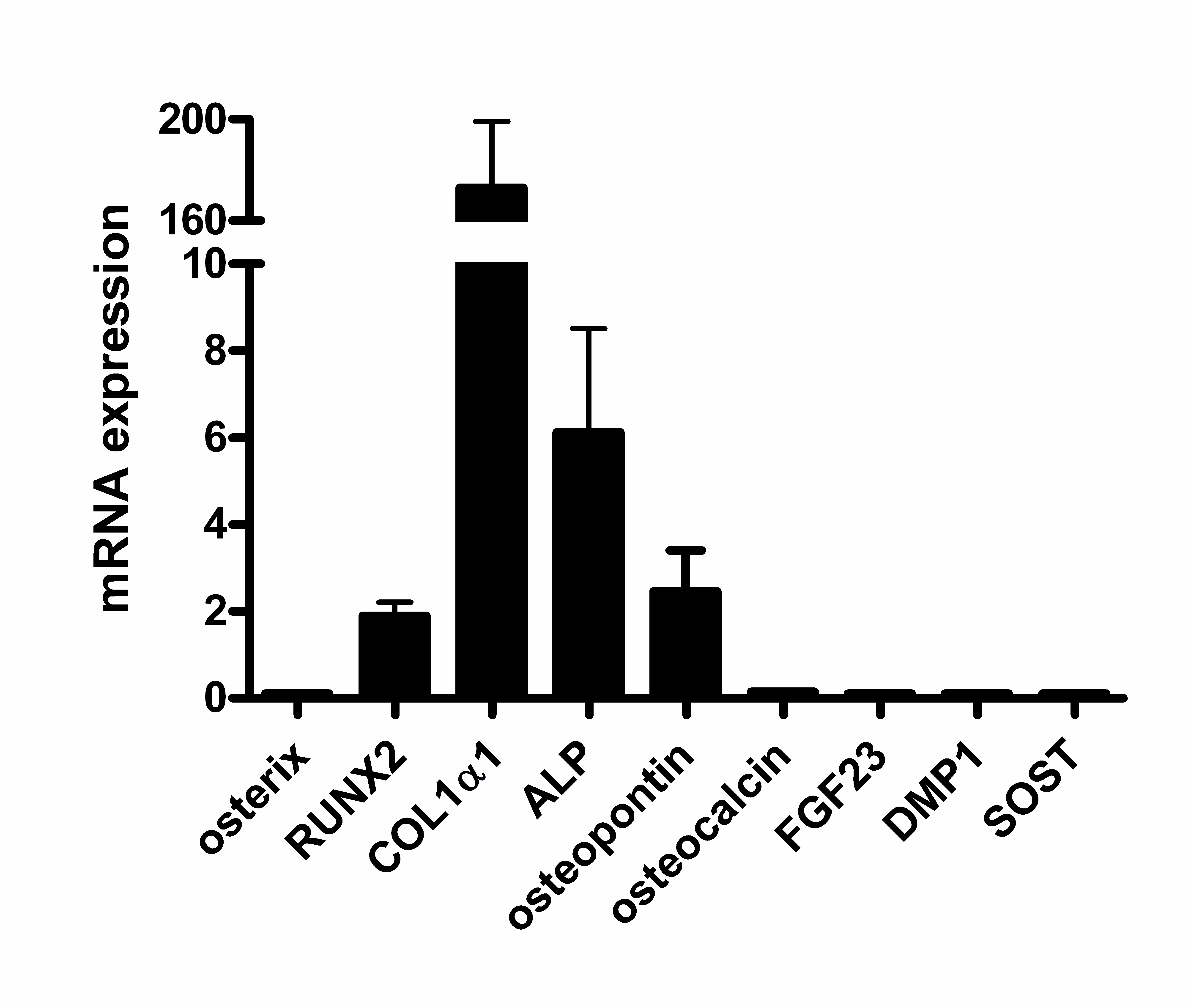
**
